# Clinical safety of home treatment for pulmonary embolism: a multicenter registry study of the Asian population

**DOI:** 10.1016/j.rpth.2025.103194

**Published:** 2025-09-23

**Authors:** Goro Yoshioka, Atsushi Tanaka, Toshiki Meguro, Masahiro Natsuaki, Yuhei Goriki, Mitsuhiro Shimomura, Keiki Yoshida, Koichi Node

**Affiliations:** 1 Department of Cardiovascular Medicine, Saga University, Saga, Japan; 2 Department of Cardiovascular Medicine, Ureshino Medical Centre, Ureshino, Japan; 3 Department of Cardiovascular Medicine, Saga Medical Centre, KOSEIKAN, Saga, Japan

**Keywords:** anticoagulation, home treatment, pulmonary embolism, safety, venous thromboembolism

## Abstract

**Background:**

The short-term safety of home-based management of pulmonary embolism (PE) in Asian patients remains unclear.

**Objectives:**

The aim of the study was to assess the 30-day clinical safety of home-based treatment compared with in-hospital treatment in Japanese patients with PE.

**Methods:**

In this multicenter retrospective study, we enrolled consecutive patients diagnosed with acute PE from the Heart and Vascular Disease Outcome Study in Saga and Kyushu Region-venous thromboembolism registry, performed in the Saga prefecture, Japan, between 2015 and 2024. Among the 641 registered patients, 104 and 537 were treated at home and in the hospital, respectively. After performing 1:1 propensity score matching, 208 patients were analyzed. The primary composite outcome was 30-day PE-related death, aggravation of venous thromboembolism, and bleeding events. Bleeding events were defined based on the Global Utilization of Streptokinase and Tissue Plasminogen Activator for Occluded Coronary Arteries criteria as moderate/severe.

**Results:**

After propensity score matching, the mean ± SD patient age was 66 ± 14 years, and 45% of the patients were men. In total, 72% of the patients had deep vein thrombosis, and 81% were treated with a direct oral anticoagulant. The primary composite outcome occurred in 3 patients (3%) in the home treatment group and in 6 patients (6%) in the in-hospital treatment group, with no significant difference between the 2 groups (log-rank test, *P* = .69). Individual components of the primary composite outcome were also comparable.

**Conclusion:**

The risk of PE-related events and bleeding in Japanese patients with PE treated at home was comparable to that of patients who received in-hospital treatment.

## Essentials

• The safety of home-based management of PE in Asian patients remains unclear.
• We compared the 30-day clinical events of home-based vs in-hospital care in Japanese patients with PE.
• After propensity score matching, the incidence of clinical events was similar between the 2 settings.
• Home-based management of PE was clinically safe in this multicenter Japanese cohort.

## 1 Introduction

Acute pulmonary embolism (PE) is one of the fatal cardiovascular diseases [1]. The severity of acute PE varies widely [2,3], and several risk stratification models are useful in detecting patients at high risk of PE-related events. Short-term hospitalization or home treatment is recommended by several guidelines for low-risk patients as a result of eligible risk stratification [4,5]. Home treatment has potential benefits in terms of patient satisfaction and reduced economic costs [5], and the trend of home treatment is on the rise [6,7].

Recent studies using PE severity to detect low-risk patients have demonstrated the clinical safety of home treatment in clinical practice [3,8]. However, previous studies on home treatment in Asian patients with PE included relatively small sample sizes and had potential bias due to differences in several clinical backgrounds between the home-based and in-hospital treatment groups [9]. Asians are at a higher risk of bleeding [10], and clinical studies specific to Asian patients with PE are required to assess the clinical safety of home treatment in this population. Therefore, the aim of the study was to investigate the short-term safety of home treatment of Japanese patients with PE using multicenter retrospective data.

## 2 Methods

### 2.1 Design and population

**Figure 1:**
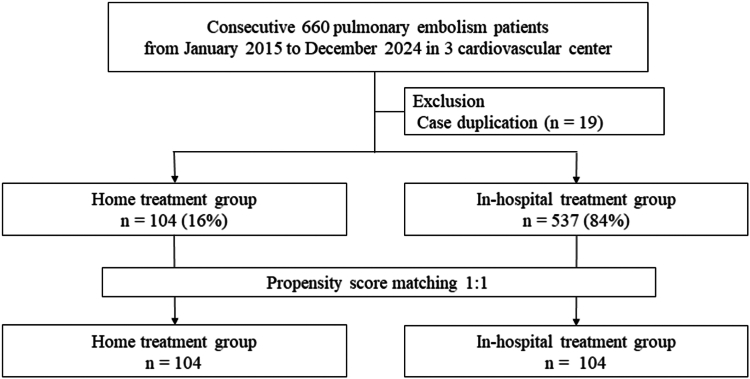
Flow diagram of the study cohort.

The Heart and Vascular Disease Outcome Study in Saga and Kyushu Region-venous thromboembolism (VTE) registry is a multicenter, nonrandomized, retrospective cohort study performed across 3 institutions in Japan. In total, 660 consecutive patients with acute PE were enrolled between January 2015 and December 2024. After excluding duplicate patients (*n* = 19), 641 patients were included in the analysis, with 104 treated at home and 537 treated in the hospital (Supplementary Table S1). After performing 1:1 propensity score (PS) matching, 208 patients (104 in each group) were analyzed (Figure 1).

This study was conducted in accordance with the ethical standards of the responsible committees on human experimentation (institutional and national) and the Helsinki Declaration of 1964, along with its later revisions. Ethics approval was obtained from the Institutional Review Board of Saga University, Saga Medical Centre KOSEIKAN, and Ureshino Medical Centre.

### 2.2 Diagnosis and treatment of PE

PE diagnosis was performed using computed tomography based on standard medical practice. Treatment strategies, including anticoagulation, thrombolysis, and the use of an inferior vena cava filter (IVCF), depended on the discretion of each physician; however, all treatments adhered to local guidelines in Japan. The decision to administer home-based or in-hospital treatment was made by the physician based on the severity of PE, bleeding risk, and other factors in accordance with Japanese guidelines.

Home treatment of PE was defined as discharge directly from the cardiovascular center within 12 hours of a PE diagnosis. All other patients were defined as in-hospital treatment, including patients with PE who were already hospitalized. The severity of PE was evaluated using the simplified version of the PE Severity Index (sPESI) [11,12], which includes the following variables: age >80 years, a history of cancer, a history of chronic cardiopulmonary disease, heart rate ≥110 beats/min, systolic blood pressure <100 mm Hg, and percutaneous arterial oxygen saturation <90%. Bleeding risk was calculated via the VTE-BLEED risk score [12], which includes age ≥60 years, active cancer, men with uncontrolled arterial hypertension, anemia, a history of bleeding, and renal dysfunction.

### 2.3 Data collection and clinical outcomes

Data were collected on baseline characteristics of patients, including medical history, vital signs, symptoms, laboratory test results, transthoracic echocardiography, electrocardiography, clinical treatment, and clinical outcomes. Clinical follow-up data were obtained through clinic visits, telephone calls, and records from hospital admissions.

The primary composite outcome was defined as 30-day PE-related death, aggravation of VTE, and bleeding events. Secondary outcomes included each component of the primary outcome. PE-related death was defined as death following clinically severe PE or death that could not be attributed to any cause other than PE. Aggravation of VTE included PE and/or deep vein thrombosis (DVT), defined as a new or worsening thrombus identified in the pulmonary arteries and deep veins on limb venous ultrasonography or computed tomography scan, with or without symptoms. Bleeding events were defined based on the Global Utilization of Streptokinase and Tissue Plasminogen Activator for Occluded Coronary Arteries (GUSTO) criteria as moderate/severe [13].

### 2.4 Statistical analysis

Normally distributed variables are presented as mean ± SD, whereas nonnormally distributed variables are presented as median and IQR. Continuous data were analyzed using an unpaired *t*-test for normally distributed data and the Wilcoxon rank-sum test for nonnormally distributed data. Categorical data were analyzed using the chi-square or Fisher’s exact test, as appropriate. All tests were 2-sided, and significance was set at *P* < .05. The cumulative incidence of outcomes was calculated using the Kaplan–Meier method, with differences evaluated using the log-rank test. PS matching was performed using a logistic regression model incorporating age, sex, the presence of active cancer, the VTE-BLEED score, and each component of the sPESI score to compare clinical characteristics between the home-based and in-hospital treatment groups. Sex was defined as biological sex (male or female) as recorded in the medical records. Moreover, 1:1 (case:control) nearest-neighbor matching without replacement was performed to identify individuals with similar PS from the 104 patients and 537 controls. Detailed patient characteristics are described in Supplementary Table S1. All analyses were performed using JMP version 17.1.0 (SAS Institute Inc).

## 3 Results

### 3.1 Patient characteristics and treatment after PS matching

The PS-matched cohort included 208 patients, consisting of 104 home-based and 104 in-hospital-treated patients; their characteristics are summarized in Table 1. The mean age ± SD was 66 ± 14 years, and 45% of the patients were men. Approximately 54% of patients had active cancer, and 7% had a history of VTE. Transient risk factors were identified in 10% of patients, while 36% had an unprovoked risk. DVT was observed in 72% of patients, and 70% were asymptomatic. Low-risk patients (sPESI = 0) comprised 26%, and 19% had a low bleeding risk (VTE-BLEED risk score < 2). Apart from laboratory data, baseline characteristics, including sPESI and VTE-BLEED risk scores (Supplementary Figure S1), were comparable between the 2 groups. The in-hospital treatment group showed lower serum albumin levels and higher white blood cell counts, platelet counts, and D-dimer. Despite slightly unstable vital signs, 9 patients received home treatment owing to personal preference and comorbidities (Supplementary Table S2).

**Table 1:** Baseline demographic after propensity score matching.

Variables	Total	Home treatment	In-hospital treatment	*P*
	(*N* = 208)	(*n* = 104)	(*n* = 104)	
Men	93 (45)	46 (44)	47 (45)	.889
Age (y)	66 ± 14	66 ± 13	65 ± 14	.69
Body mass index (kg/m^2^)	23 ± 5	23 ± 5	23 ± 5	.549
Active cancer	113 (54)	54 (52)	59 (57)	.486
History of VTE	14 (7)	5 (5)	9 (9)	.265
Chronic cardiopulmonary disease	34 (16)	17 (16)	17 (16)	1.00
Steroid use	16 (7)	10 (10)	6 (6)	.296
WBC (×10^3^/mL)	7.9 ± 5.0	6.7 ± 3.1	9.1 ± 6.1	<.001
Hemoglobin (g/dL)	12 ± 2	12 ± 2	12 ± 2	.18
Platelet (×10^4^/μL)	24 ± 11	22 ± 8	26 ± 13	.015
Serum albumin (g/dL)	3.4 ± 0.7	3.7 ± 0.5	3.1 ± 0.7	<.001
eGFR (mL/min/1.73m^2^)	71 ± 23	69 ± 22	72 ± 23	.37
D-dimer (μg/mL)	5.3 (2.4-12.1)	4.5 (1.9-9.9)	6.7 (2.9-14.6)	.01
VTE risk				
Transient	20 (10)	9 (9)	11 (11)	.58
Cancer	113 (54)	54 (52)	59 (57)	
Unprovoked	75 (36)	41 (39)	34 (33)	
DVT	149 (72)	72 (69)	77 (74)	.44
Asymptomatic	145 (70)	72 (69)	73 (70)	.88
PE severity				
Systolic BP < 100 mm Hg	3 (1)	2 (2)	1 (1)	.557
Pulse rate ≥ 110/min	7 (3)	4 (4)	3 (3)	.70
SpO2 < 90%	6 (3)	3 (3)	3 (3)	1.00
sPESI	1 (1-0)	1 (1-0)	1 (1-0)	.77
sPESI score < 1	55 (26)	27 (26)	28 (27)	.875
VTE-BLEED score	3.5 (2.5-5)	3.5 (2-5)	3.5 (3-5)	.497
VTE-BLEED score < 2	40 (19)	23 (22)	17 (16)	.19

Data for categorical variables are presented as *n* (%); data for continuous variables are presented as mean ± SD for normal distributions or median (IQR) for skewed distributions.

BP, blood pressure; DVT, deep vein thrombosis; eGFR, estimated glomerular filtration rate; PE, pulmonary embolism; sPESI, simplified Pulmonary Embolism Severity Index; SpO2, percutaneous arterial oxygen saturation; VTE, venous thromboembolism; WBC, white blood cell.

Initial treatment regimens for PE are summarized in Table 2. Direct oral anticoagulants (DOACs) were administered to 81% of patients. Thrombolysis was performed in only 2 patients. IVCF was used in 7 patients. One patient with an IVCF who was treated at home was a 61-year-old female with active cancer. This patient underwent IVCF placement in the outpatient setting immediately after diagnosis of PE and went home at her strong request. All treatments were comparable between the 2 groups.

**Table 2:** Treatment and clinical outcomes after propensity score matching.

Variables	Total	Home treatment	In-hospital treatment	*P*
	(*N* = 208)	(*n* = 104)	(*n* = 104)	
Treatment				
Warfarin	23 (11)	9 (9)	14 (13)	.288
DOAC	169 (81)	86 (83)	83 (80)	
Apixaban	62 (30)	26 (25)	36 (35)	.168
Edoxaban	75 (36)	40 (38)	35 (34)	
Rivaroxaban	32 (15)	20 (19)	12 (12)	
Thrombolysis	2 (1)	0	2 (2)	.155
Open heart surgery	0	0	0	
IVCF use	7 (3)	1 (1)	6 (6)	.05
30-day outcomes				
Primary composite outcome	9 (4)	3 (3)	6 (6)	.30
PE-related death	2 (1)	0	2 (2)	.155
Aggravation of VTE	1 (1)	0	1 (1)	.238
Bleeding event	6 (3)	3 (3)	3 (3)	1.00
All-cause death	7 (3)	1 (1)	6 (6)	.04

Data for categorical variables are presented as *n* (%); data for continuous variables are presented as mean ± SD for normal distributions or median (IQR) for skewed distributions.

DOAC, direct oral anticoagulation; IVCF, inferior vena cava filter; PE, pulmonary embolism; VTE, venous thromboembolism.

### 3.2 Outcomes

**Figure 2:**
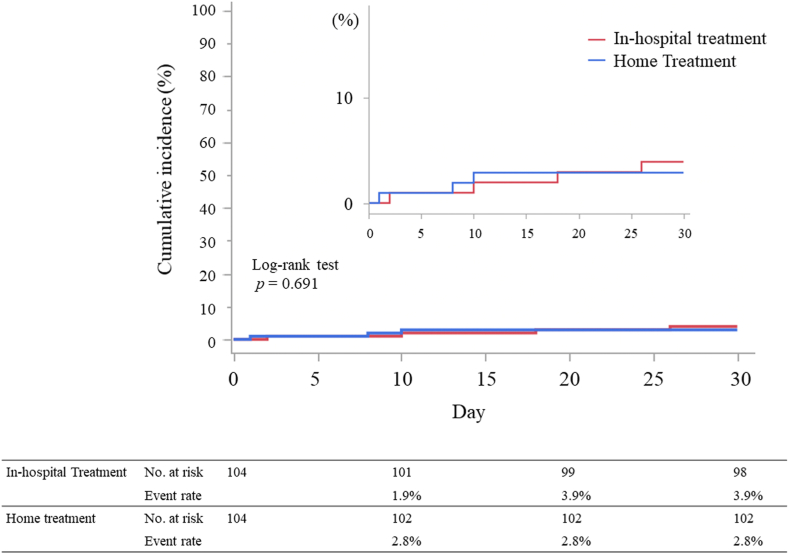
Thirty-day primary composite outcome after propensity score matching. The Kaplan‒Meier curve at 30 days shows clinical events in both the home-based and in-hospital treatment groups, with comparisons evaluated using the log-rank test.

Clinical outcomes are summarized in Table 2. The primary composite outcome occurred in 9 patients (4%): 3 of the 104 patients (3%) in the home treatment group and 6 of the 104 patients (6%) in the in-hospital treatment group. The Kaplan‒Meier curve at 30 days showed a similar cumulative incidence of the primary composite outcome (log-rank test, *P* = .69; Figure 2). These results were consistent with the pre-PS matching across all the cohorts (Supplementary Figure S2). PE-related death and aggravation of VTE within 30 days occurred in 2 of 208 patients and 1 of 208 patients, respectively. No patients with PE-related death or aggravation of VTE were observed in the home treatment group (Table 2). Bleeding events within 30 days occurred in 6 of 208 patients, with 3% and 6% (3/104) in the home-based and in-hospital treatment groups, respectively. Bleeding events in the in-hospital treatment group included 2 patients with gastrointestinal bleeding and 1 patient with urinary tract bleeding. Bleeding events in the home treatment group included 1 patient with gastrointestinal bleeding, 1 patient with gynecological bleeding, and 1 patient with unknown anemia. Individual components of the primary composite outcome were also comparable. All-cause death within 30 days occurred in 7 patients (4%), and the in-hospital treatment group exhibited a higher event rate than the home treatment group (1% vs 6%; *P* = .04).

### 3.3 Subgroup analysis of patients with or without active cancer

The clinical backgrounds of patients with and without active cancer are summarized in Supplementary Tables S3 and S4. Except for laboratory data, all patient characteristics were comparable between the 2 groups. The in-hospital treatment group showed lower serum albumin levels and higher white blood cell counts. Clinical outcomes are summarized in Supplementary Tables S5 and S6. In patients with active cancer, the primary composite outcome occurred in 8 patients (7%): 3 of the 54 patients (6%) in the home treatment group and 5 of the 59 patients (8%) in the in-hospital treatment group (*P* = .54). In patients without active cancer, the primary composite outcome occurred in only 1 patient (1%) in the in-hospital treatment group; no events occurred in the home treatment group. Regardless of the presence of active cancer, the 30-day event rate in the home treatment group was similar to that of the in-hospital group.

## 4 Discussion

There were several major findings in this study. First, this multicenter retrospective study of Asian patients with PE showed that 16% of the patients (104/641) received home treatment in a real-world clinical setting. Second, after PS matching, home treatment for PE showed short-term safety with respect to the composite outcome of PE-related death, aggravation of VTE, and bleeding events. Thus, even in this Asian cohort, home treatment for PE was not associated with a higher incidence of bleeding events and was safe for patients with and without active cancer.

The number of patients with VTE is increasing owing to advances in diagnosis and improved prognosis in patients with cancer [9]. Acute PE, a form of VTE, is also increasing [2] and is becoming a common disease in daily clinical practice, often managed by noncardiologists. Several risk models have identified patients at high risk of PE-related events and are used in routine clinical practice [14]. The PE Severity Index and sPESI scores are well-established tools, and their utility in predicting all-cause mortality within 1 month has been demonstrated [15]. The Hestia rule is also an effective risk model [16], and both the Hestia rule and sPESI strategies have shown similar safety and effectiveness [17]. Guidelines, including the European Society of Cardiology and the Japanese Circulation Society, recommend assessing the sPESI class as part of safe clinical practice in patients with PE. These guidelines also suggest short-term hospitalization or home treatment for low-risk patients based on the sPESI class [4,18].

Currently, home treatment is generally recommended for low-risk patients with PE [5], provided that their home circumstances are appropriate for care. Between 1990 and 1995, home treatment of DVT was safe, effective, and cost-efficient for carefully selected patients [19]. Similarly, home treatment of patients with PE may offer greater patient satisfaction and economic cost benefits than in-hospital patient care [3]. In the DOAC era, which offers easier management than warfarin, the trend of home treatment is on the rise [6,7]. DOACs have overtaken warfarin as the primary outpatient treatment for VTE, with 65% to 82% of patients reported using DOACs instead of warfarin between 2012 and 2017 [20]. Recent meta-analyses using PE severity to identify low-risk patients have demonstrated the clinical safety of home treatment [21]. Another study on the home treatment of low-risk patients with PE in the DOAC era have demonstrated that the home treatment group (*n* = 1814) had 90-day major adverse outcomes rates below 1%, including all-cause mortality (0.7%; 95% CI, 0.4%-1.2%), PE-related mortality (0.06%; 95% CI, 0.01%-0.3%), and recurrent VTE (0.8%; 95% CI, 0.5%-1.4%) [22].

However, even in the DOAC era, bleeding events remain an important concern. In patients with VTE, major bleeding occurred in 8% of the total cohort (median follow-up period of 1218 days) and was associated with a subsequent mortality risk [23]. In patients with VTE and active cancer, the 5-year incidence of major bleeding events following warfarin treatment was 26.6% [24], and high event rates (20.4%) have also been reported in the DOAC era [25].

Asian patients who are at high risk of bleeding [26] require careful risk stratification when considering home treatment. A prospective study on home treatment of Asian patients with PE demonstrated its safety [9]. However, in this study, potential bias was also raised due to differences in several clinical backgrounds between the home-based and in-hospital treatment groups. Therefore, we aimed to investigate the safety of home treatment of Asian patients with PE using multicenter retrospective data and PS matching.

This study revealed that in a multicenter registry of Asian patients with PE, 16% (104/641 patients) received home treatment in a daily clinical setting. After PS matching, home treatment for PE demonstrated safety regarding the composite outcome of PE-related death, aggravation of VTE, and bleeding events. Even in an Asian cohort with PE, home treatment was not associated with a higher incidence of bleeding events. Subgroup analysis of patients with active cancer also demonstrated that the safety of the home treatment strategy was consistent across all groups.

The home treatment strategy for Asian patients has not been well established owing to insufficient clinical data. Even in patients with a sPESI score of 0, home treatment was infrequently applied between 2015 and 2020 in Japan [3]. Chatani et al. [9] recently reported that patients with active cancer and an sPESI score of 1, based on data from a Japanese randomized controlled trial, could be potential candidates for home treatment. Our results suggest the possibility of expanding home treatment to active cancer cases. Although the data were derived from a randomized controlled trial, post hoc analysis revealed significant differences in vitals, treatment, and other factors between the home-based and in-hospital groups. To establish a home treatment strategy for Asian populations, further accumulation of clinical evidence is still warranted. In this context, our findings provide additional clinical evidence on home treatment for PE, adjusted for baseline characteristics using PS matching. In addition, the determination of in-hospital or home treatment, the severity of comorbidities, and social background were also considered.

The decision to treat at home or in hospital is made based on the patient’s risk status and the physician’s judgment in real-world clinical practice. Given that all events that occurred in the home treatment group in this study were observed in patients with active cancer, appropriate risk assessment using other risk models, such as the VTE-BLEED score, would be needed for patients with active cancer. ''In addition, when considering home treatment for patients with high-risk scores, it is desirable to carefully obtain detailed informed consent and provide guidance on how to respond to adverse events (eg, appropriate methods for accessing medical care).

Even after PS matching, the incidence of the primary outcome was numerically, but not significantly, higher in the in-hospital group than in the home treatment group. The general risk of VTE and bleeding events was adjusted through PS matching; nonetheless, other indicators, such as white blood cell count, serum albumin levels, and D-dimer, were worse in the in-hospital treatment group. These data might reflect the poor general condition of patients in the in-hospital treatment group. In addition, the incidence of all-cause mortality in the in-hospital treatment group was significantly higher than that in the home treatment group. Most deaths were non–PE-related and caused by underlying diseases, particularly cancer progression. The prevalence of active cancer was comparable between the 2 groups; nevertheless, detailed data on active cancer, such as site and staging, were lacking and potentially affected the study outcomes. These reasons might be associated with the increased incidence of adverse events in the in-hospital treatment group.

### 4.1 Limitations

This study had some limitations. First, this was a retrospective observational study conducted in Japanese centers. Our findings require careful consideration when applying them to other populations. However, it is well-known that Asian populations have different bleeding risks compared with non-Asian populations. Few studies have used PS matching in Asian populations, and there are clinical implications for assessing the safety of home treatment in this population. Second, treatment strategies were not predefined and were generally performed based on local treatment guidelines. In addition to the sPESI score and bleeding risk, the severity of comorbidities and social background were also considered when determining where patients should be treated. Hence, the home treatment group also included patients with sPESI ≥1 or a VTE-BLEED risk score ≥2. These factors may have influenced the study outcomes. Nonetheless, our findings, obtained from real-world cohort data, may be useful in decision-making for patients with PE in daily clinical practice. Third, data on the type, progression, and treatment of malignant tumors are lacking. The stage of malignancy should be considered, as it influences the treatment strategy and prognosis of patients with PE. However, since this study focuses on short-term prognosis as an endpoint, the impact of this limitation is minimized. Fourth, data on N-terminal pro-brain natriuretic peptide, troponin, and right ventricular function are lacking. The inclusion of right ventricular dysfunction and biomarkers could further improve risk stratification capabilities [17,27]. Fifth, although PS matching was conducted, significant group differences were observed in the laboratory data. These differences might be associated with the poor general condition of the in-hospital treatment group. Sixth, the absence of low-molecular-weight heparin use in Japan may influence the results of this study. Finally, we defined a bleeding event based on the GUSTO criteria as moderate/severe, rather than using the major bleeding criteria defined by the Bleeding Academic Research Consortium (ie, 3 or 5). However, the rates of major bleeding were similar regardless of the definition, ie, GUSTO moderate/severe or Bleeding Academic Research Consortium 3 or 5, as reported previously [28].

## 5 Conclusions

The risk of PE-related events and bleeding in Japanese patients with PE treated at home was comparable to that in those treated in-hospital. Nationwide real-world evidence is warranted to further validate the safety of outpatient treatment for PE.
