# Supplementary material for: Clinical safety of home treatment for pulmonary embolism: a multicenter registry study of the Asian population
Source: Res Pract Thromb Haemost. 2025 Sep 23;9(7):103194. doi: 10.1016/j.rpth.2025.103194 (PMC12554170; doi:10.1016/j.rpth.2025.103194)
Supplement: Supplementary Material [file mmc1.pdf]

Supplementary Table S1. Baseline demographic and clinical characteristics of the entire cohort

Variables	Total (n = 641)	Home treatment (n = 104)	In-hospital treatment (n = 537)	*p*
Man, n (%)	245 (38)	46 (44)	199 (37)	0.171
Age (years)	67 ± 15	66 ± 13	68 ± 15	0.391
Body mass index (kg/m^2)	23 ± 5	23 ± 5	23 ± 5	0.975
Active cancer, n (%)	235 (37)	54 (52)	181 (34)	<0.001
History of VTE, n (%)	29 (4)	5 (5)	24 (4)	0.880
Chronic cardiopulmonary disease, n (%)	60 (9)	17 (16)	43 (8)	0.013
Steroid use, n (%)	48 (8)	10 (10)	38 (7)	0.391
WBC (×10^3/mL)	8.5 ± 5.0	6.7 ± 3.1	8.9 ± 5.2	<0.001
Hemoglobin (g/dL)	12 ± 2	12 ± 2	12 ± 3	0.155
Platelet (×10^4/μL)	23 ± 10	22 ± 8	23 ± 11	0.193
Serum albumin (g/dL)	3.2 ± 0.7	3.7 ± 0.5	3.1 ± 0.7	<0.001
eGFR (mL/min/1.73m^2)	71 ± 25	69 ± 22	72 ± 27	0.321
D-dimer (μg/mL)	8.4 (4.0–7.9)	4.5 (1.9–9.9)	9.2 (4.6–19.6)	0.013
VTE risk, n (%)				
Transient	173 (27)	9 (9)	164 (31)	
Cancer	235 (36)	54 (52)	181 (33)	<0.001
Unprovoked	233 (367)	41 (39)	192 (36)	
DVT, n (%)	485 (76)	72 (69)	413 (77)	0.102
Asymptomatic, n (%)	340 (53)	72 (69)	268 (50)	<0.001
PE severity, n (%)				
Systolic BP < 100 mmHg	44 (7)	2 (2)	42 (8)	0.013
Pulse rate ≥ 110 /min	75 (12)	4 (4)	71 (13)	0.002
SpO2 < 90 %	131 (20)	3 (3)	128 (24)	<0.001
sPESI score < 1	184 (29)	27 (26)	157 (29)	<0.001
VTE-BLEED risk score < 2	174 (27)	23 (22)	151 (28)	0.199

Data for categorical variables are presented as number (%); data for continuous variables are presented as mean ± standard deviation for normal distribution or median (interquartile range) for skewed distribution.

DVT, deep venous thrombosis; eGFR, estimated glomerular filtration rate; PE, pulmonary embolism; sPESI, simplified pulmonary embolism severity index; VTE, venous thromboembolism; WBC, white blood cell.

Supplementary Table S2. List of patients with unstable vitals in the home treatment group

	Vital status	Age	Sex	Active cancer	sPESI score	Clinical features
Case 1	SBP 98 mmHg	56	Male	Yes	2	Refusal of in-hospital care due to terminal stage of cancer
Case 2	SBP 99 mmHg	69	Female	No	2	Refusal of in-hospital care due to personal preference
Case 3	HR 127 /min	57	Male	No	2	Tachycardia due to acute pneumoniae
Case 4	HR 128 /min	49	Female	Yes	2	Refusal of in-hospital care due to personal preference
Case 5	HR 115 /min	48	Female	Yes	2	Refusal of in-hospital care due to personal preference
Case 6	HR 112 /min	63	Male	Yes	2	Refusal of in-hospital care due to schizophrenia
Case 7	Under oxygen therapy	75	Male	Yes	2	Refusal of in-hospital care due to terminal stage of cancer
Case 8	Under oxygen therapy	85	Male	No	3	Oxygen administration for COPD
Case 9	Under oxygen therapy	96	Female	No	2	Oxygen administration for unknown lung disease

COPD, chronic obstructive pulmonary disease, HR; hear rate; SBP, systolic blood pressure; sPESI, simplified pulmonary embolism severity index

Supplementary Table S3. Baseline demographic and clinical characteristics of patients with active cancer

Variables	Total (n = 113)	Home treatment (n = 54)	In-hospital treatment (n = 59)	*p*
Man, n (%)	50 (44)	23 (43)	27 (46)	0.735
Age (years)	65 ± 12	64 ± 12	66 ± 12	0.495
Body mass index (kg/m^2)	23 ± 5	23 ± 5	23 ± 5	0.861
Active cancer, n (%)	113 (100)	54 (100)	59 (100)	
History of VTE, n (%)	9 (8)	4 (7)	5 (8)	0.834
Chronic cardiopulmonary disease, n (%)	7 (6)	2 (4)	5 (8)	0.285
Steroid use, n (%)	3 (3)	2 (4)	1 (2)	0.504
WBC (×10^3/mL)	8.0 ± 6.1	6.7 ± 3.6	9.3 ± 7.7	0.027
Hemoglobin (g/dL)	12 ± 2	12 ± 4	11 ± 3	0.602
Platelet (×10^4/μL)	24 ± 10	23 ± 4	26 ± 13	0.119
Serum albumin (g/dL)	3.4 ± 0.7	3.7 ± 0.4	3.1 ± 0.7	<0.001
eGFR (mL/min/1.73m^2)	70 ± 21	69 ± 22	71 ± 20	0.556
D-dimer (μg/mL)	5.2 (1.9–14.7)	4.3 (1.7–11.5)	6.5 (1.9–18.3)	0.115
VTE risk, n (%)				
Transient	0	0	0	
Cancer	113 (100)	54 (100)	59 (100)	
Unprovoked	0	0	0	
DVT, n (%)	80 (71)	41 (76)	39 (66)	0.250
Asymptomatic, n (%)	83 (73)	39 (72)	44 (75)	0.777
PE severity, n (%)				
Systolic BP < 100 mmHg	2 (2)	1 (2)	1 (2)	0.950
Pulse rate ≥ 110 /min	5 (4)	3 (6)	2 (3)	0.576
SpO2 < 90 %	2 (2)	1 (2)	1 (2)	0.950
sPESI score < 1	0	0	0	
VTE-BLEED risk score < 2	0	0	0	

Data for categorical variables are presented as number (%); data for continuous variables are presented as mean ± standard deviation for normal distribution or median (interquartile range) for skewed distribution.

DVT, deep venous thrombosis; eGFR, estimated glomerular filtration rate; PE, pulmonary embolism; sPESI, simplified pulmonary embolism severity index; VTE, venous thromboembolism; WBC, white blood cell.

Supplementary Table S4. Baseline demographic and clinical characteristics of patients without active cancer

Variables	Total (n = 95)	Home treatment (n = 50)	In-hospital treatment (n = 45)	*p*
Man, n (%)	43 (45)	23 (46)	20 (44)	0.879
Age (years)	67 ± 16	68 ± 15	65 ± 17	0.312
Body mass index (kg/m^2)	23 ± 6	24 ± 6	23 ± 6	0.524
Active cancer, n (%)	0	0	0	
History of VTE, n (%)	5 (5)	1 (2)	4 (9)	0.123
Chronic cardiopulmonary disease, n (%)	27 (28)	15 (30)	12 (27)	0.719
Steroid use, n (%)	13 (14)	8 (16)	5 (11)	0.487
WBC ($\times 10^3$/mL)	7.7 ± 3.2	6.6 ± 2.6	8.8 ± 3.4	0.001
Hemoglobin (g/dL)	12 ± 2	13 ± 2	12 ± 2	0.192
Platelet ($\times 10^4$/μL)	23 ± 11	21 ± 8	25 ± 14	0.075
Serum albumin (g/dL)	3.4 ± 0.7	3.6 ± 0.5	3.1 ± 0.7	<0.001
eGFR ($mL/min/1.73m^2$)	71 ± 24	70 ± 21	73 ± 27	0.488
D-dimer (μg/mL)	5.6 (2.9–10.7)	4.9 (2.3–9.3)	6.8 (3.4–6.8)	0.141
VTE risk, n (%)				
Transient	20 (21)	9 (18)	11 (24)	
Cancer	0	0	0	0.442
Unprovoked	75 (79)	41 (82)	34 (76)	
DVT, n (%)	69 (73)	31 (62)	38 (84)	0.014
Asymptomatic, n (%)	62 (65)	33 (66)	29 (64)	0.874
PE severity, n (%)				
Systolic BP < 100 mmHg	1 (1)	1 (2)	0	1.000
Pulse rate ≥ 110 /min	1 (2)	1 (2)	1 (2)	1.000
SpO2 < 90 %	4 (4)	2 (4)	2 (4)	1.000
sPESI score < 1	40 (42)	23 (46)	17 (38)	0.417
VTE-BLEED risk score < 2	55 (58)	27 (54)	28 (62)	0.418

Data for categorical variables are presented as number (%); data for continuous variables are presented as mean ± standard deviation for normal distribution or median (interquartile range) for skewed distribution.

DVT, deep venous thrombosis; eGFR, estimated glomerular filtration rate; PE, pulmonary embolism; sPESI, simplified pulmonary embolism severity index; VTE, venous thromboembolism; WBC, white blood cell.

Supplementary Table S5. Treatment and clinical outcomes of patients with active cancer

Variables	Total (n = 113)	Home treatment (n = 54)	In-hospital treatment (n = 59)	*p*
Treatment				
Warfarin, n (%)	11 (10)	7 (13)	4 (7)	0.411
DOAC, n (%)	93 (82)	46 (85)	47 (80)	0.408
Apixaban	42 (38)	18 (33)	24 (42)	
Edoxaban	36 (32)	19 (35)	17 (30)	0.506
Rivaroxaban	15 (14)	9 (17)	6 (11)	
Thrombolysis, n (%)	0	0	0	
Open heart surgery, n (%)	0	0	0	
IVCF use, n (%)	3 (3)	1 (2)	2(3)	0.607
30-day outcome				
Composite outcome, n (%)	8 (7)	3 (6)	5 (8)	0.543
PE-related death	2 (2)	0	2 (3)	0.172
Worsening of VTE	0	0	0	
Bleeding event	6 (5)	3 (6)	3 (5)	0.913
All cause death, n (%)	6 (5)	1 (2)	5 (8)	0.117

Data for categorical variables are presented as number (%); data for continuous variables are presented as mean ± standard deviation for normal distribution or median (interquartile range) for skewed distribution.

DOAC, direct oral anticoagulation; IVCF, inferior vena cava filter; PE, pulmonary embolism; VTE, venous thromboembolism

Supplementary Table S6. Treatment and clinical outcomes of patients without active cancer

**Variables**	**Total (n = 95)**	**Home treatment (n = 50)**	**Hospitalization (n = 45)**	***p***
Treatment				
Warfarin, n (%)	12 (12)	5 (10)	7 (16)	0.480
DOAC, n (%)	76 (80)	40 (80)	36 (80)	0.480
Apixaban	20 (21)	8 (16)	12 (27)	
Edoxaban	39 (41)	21 (43)	18 (40)	0.461
Rivaroxaban	17 (18)	11 (22)	6 (13)	
Thrombolysis, n (%)	2 (2)	0	2 (4)	0.132
Open heart surgery, n (%)	0	0	0	
IVCF use, n (%)	4 (4)	0	4(9)	0.031
30-day outcome				
Composite outcome, n (%)	1 (1)	0	1 (2)	0.289
PE-related death	0	0	0	
Worsening of VTE	1 (1)	0	1 (2)	0.289
Bleeding event	0	0	0	
All cause death, n (%)	1 (1)	0	1 (2)	0.289

Data for categorical variables are presented as number (%); data for continuous variables are presented as mean ± standard deviation for normal distribution or median (interquartile range) for skewed distribution.

DOAC, direct oral anticoagulation; IVCF, inferior vena cava filter; PE, pulmonary embolism; VTE, venous thromboembolism

Supplementary Figure S1. Distribution of the sPESI and VTE-BLEED scores in each treatment group

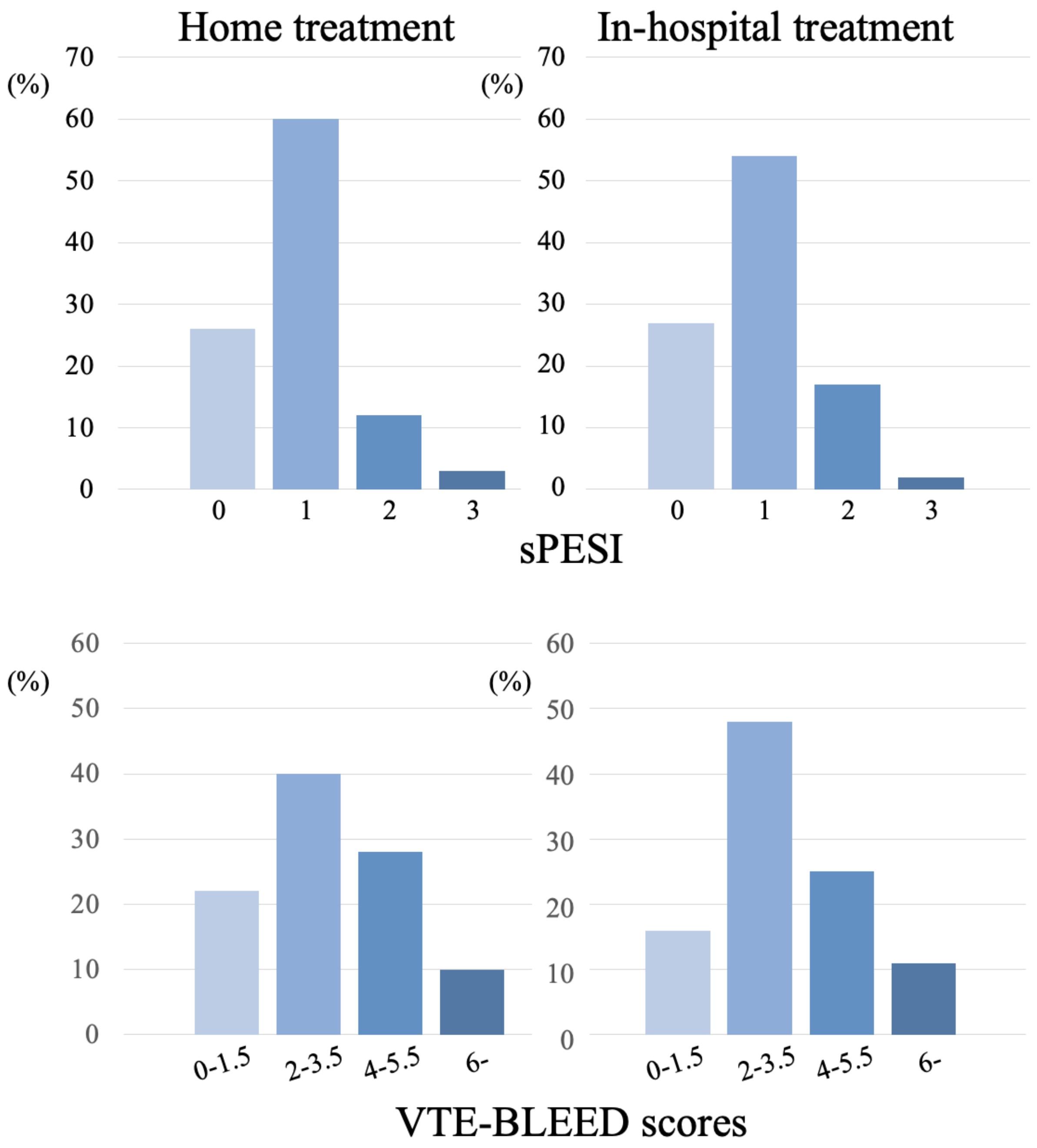

sPESI, simplified pulmonary embolism severity index

Supplementary Figure S2. 30-day primary composite outcome in all cohort

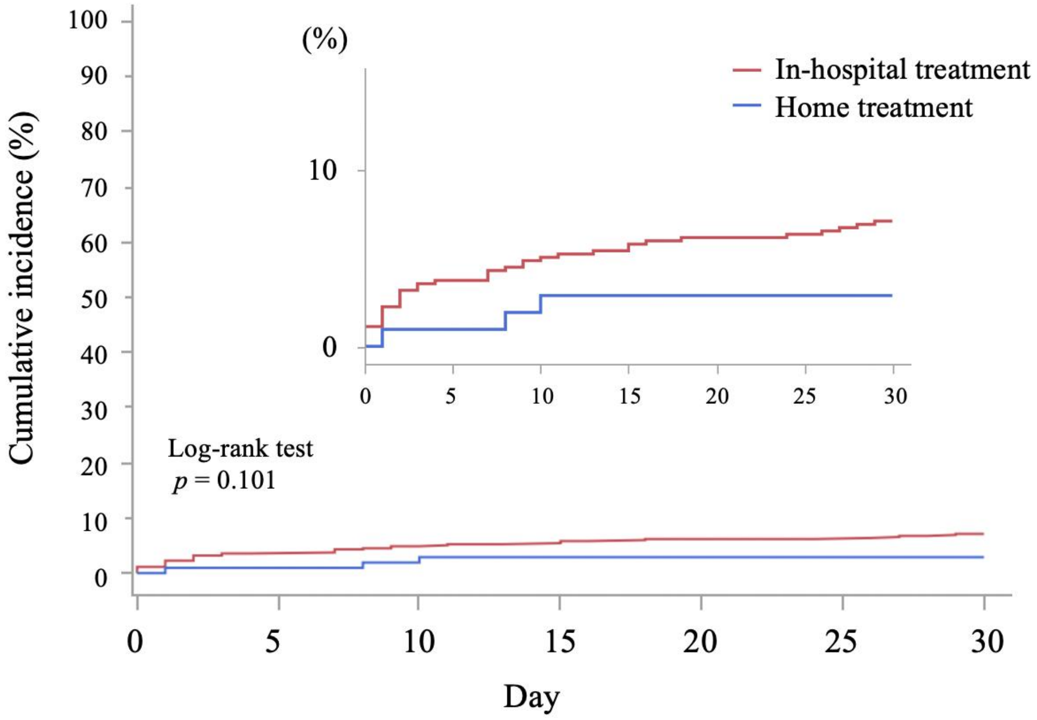

In-hospital Treatment	No. at risk	537	508	501	496
	Event rate		5.0%	6.4%	11.1%
Home treatment	No. at risk	104	102	102	102
	Event rate		2.8%	2.8%	2.8%
